# Homœopathy as It Was and Is

**Published:** 1871-09

**Authors:** Charles W. Earle


CHICAGO

MEDICAL EXAMINER:

N. S. DAVIS, M.D., EDITOR.

F. H. DAVIS, M.D., ASSISTANT-EDITOR.

VOL. XII. SEPTEMBER, 1871. NO. 9.

### Original Contributions.

### HOMŒOPATHY AS IT WAS AND AS IT IS.

A Paper Read before the Chicago Medical Society, July 17, 1871.

By CHARLES W. EARLE, M.D.

[PUBLISHED BY REQUEST.]

MR. PRESIDENT AND GENTLEMEN:—One year ago my term of hospital service expired, and I commenced the practice of medicine. During this short time I have not failed to notice the difference of opinion among the older, learned, and distinguished practitioners, as to the policy of attacking homœopathy.

I do not claim to have discovered the *correct* policy, and would never urge this as the reason for presenting this article.

I write upon this subject, gentlemen, believing that by so doing I shall myself become better acquainted with the merits or demerits of the so-called system, and with the hope that I may furnish to my younger brethren—colleagues in the regular profession—the correct status, as far as it is possible for me to learn, of homœopathy.

I shall not attempt to amuse, by telling stories of how babies have swallowed large quantities of homœopathic granules—highly attenuated, it may be—of course, *powerful*—and still live; nor do I wish to abuse, or even speak ill, of any gentleman who may practice homœopathy, either because he can make more money by it, or is conscientious in doing so.

With these views it is certainly proper for me to write on this subject.

It appears to me also, that the medical profession, if they believe homœopathy a humbug, have the same right to speak to the people in regard to it, to warn them against it, to explain the false system to them, as the members of any other profession. The ministers of the gospel do this—they stand in the pulpit and explain to us the fallacies in theology that carry away a certain part of the people—the irreligions, that, as it seems to them, are sapping the morals of the community. But I do not at present believe it a humbug; I will not, unless, indeed, I *prove* it.

Let us now examine this system. What I have to present can best be arranged under the following distinct heads:

1st. Homœopathy as practiced by Hahnemann.

2d. The attempts made by homœopathists to foist the system into public favor, with the experiments made by different governments.

3d. Homœopathy as now practiced.

4th. A brief notice of Grauvogl's recent work.

Samuel Hahnemann, a native of Saxony, was born in 1755. When about the age of twenty years he commenced the study of medicine at Leipzic. During his studentship he was certainly not distinguished for his remarkable application or interest in his chosen profession.

Very early he evinced a susceptibility of being led away by transcendental follies, and in less than three years became a follower of Mesmer, or, at least, influenced by his teachings. It appears to us, also, that he lacked *stability* of purpose, that great essential for a successful medical man. That he lacked this essential will be suspected, when the fact is made known to us, that in twenty-eight years he changed his residence no less than twenty-four times; and also, during these years, he taught at one time German and Latin—at another time devoted himself to chemistry and botany—had charge of an insane asylum—embraced Mesmerism, etc., etc.

He does not seem to have been a person whom we would naturally expect to have been a leader in his profession—much less, one to discover a new system of medicine—able and destined to supercede a system which had steadily advanced side by side with the other kindred sciences for centuries. But in 1810, he commenced to lecture—gave to his own system a classical name, and gratuitously called those practising the regular profession *Allopaths.* The rapidity with which he perfected the system is truly remarkable. He speaks of his great method of cure as "infallible," "an unerring law," and as "the great sole therapeutic law."

In these days, with facilities acknowledged to be far superior to those enjoyed at the commencement of the century, the most ambitious student would be content, at the end of his labors, with one-hundredth part of what Hahnemann claimed at his first lecture. The substance of Hahnemann's theory, and what he claimed for it, is very well understood by you, and I will not here enter into any elaborate elucidation. All existing medical knowledge was claimed to be false. The ordinary practice of medicine was causing the death of many of those who were being treated by it, and when death did not ensue, the persons treated were left in a horrible condition, caused by the poisonous medicine. Diseases were to be cured by remedies capable of producing symptoms resembling those found in the disease under treatment. Medicine is powerful only when reduced to a wonderful degree of minuteness. Nature effects but little in the cure of disease. Only one medical substance should be administered at the same time. The union of several drugs destroys the effect, and sometimes produce a new disease. Many substances, commonly inert, by great shaking and diluting, develop great power.

The last theory of Hahnemann's which I shall enumerate, and perhaps the most startling, is that *seven-eighths* of all diseases are caused by Psora. Startling as it may be to us, Hahnemann declares that nervous debility, hysteria, insanity, madness, epilepsy, rickets, cancer, yellow jaundice, gastralgia, epistaxis, hæmoptysis, asthma, deafness, and all other kinds of pain are caused by Psora, or, in the language of the non-scientific, that little harmless, and to some, comfortable disease—the itch. And it took Hahnemann twelve years to establish this one fact. After all this labor it does seem cruel that any body can be found who does not believe it.

I have thus given several leading ideas as advanced by Hahnemann, and professedly believed by his followers. Very many, both scientific and non-scientific people, would not care to investigate further a subject with so many pretensions and evident fallacies. They would dismiss it at once as unworthy of close investigation. Suppose a man should submit to the Academy of Sciences in this city, a paper in which he claimed that he had traveled to the moon, had actually conversed with the inhabitants there, and would now enlighten the Academy on the subject. Would they give it a thought? Suppose a person should say to the Faculty of the University at Evanston, I can square a circle—I can demonstrate it. Would they spend their time listening to him? There are certain things which are *absurd*—people let them alone.

But people have believed the system of Hahnemann, and so I continue my investigation. Let us be thorough and examine the details, and, first, how did Hahnemann prepare his medicine?

In order to appreciate the minuteness and delicacy of preparation, I quote from Hahnemann's *Organon*, 2d Am. Ed., p. 200: "If two drops of a mixture of equal parts of alcohol and recent juice of any medicinal plant be diluted with ninety-eight drops of alcohol, in a vial capable of containing 130 drops (for convenience of shaking), and the whole twice shaken together, the medicine becomes excited in energy to the first development of power, or, as it may be denominated, the first potence.

"The process is to be continued through twenty-nine additional vials. * * * * *

"These manipulations are to be conducted thus through all the vials, from the first up to the thirtieth, or decillionth development of power which is the one in most general use."

The mode of preparation of medicine in the form of powder is quite similar, and needs not a description.

THE DOSE.—Hahnemann recommended the use of small globules of sugar, of the size of mustard seed; one of these globules forms a dose, containing about a three-hundredth-part of a drop of the dilution. Several years after he recommended that the globules should be merely smelled, and observed (see *Organon*, p. 332), that " all that Homœopathy is capable of curing, * * will be most safely and certainly cured by this mode of olfaction." Testimony is not wanting to prove the power of his olfaction. A certain doctor, one of his disciples, says: "My own wife was cured by him, in this manner, of a violent pleurisy in the course of five hours." Men and women walk the streets of our city to-day who certainly profess to believe that medicine, thus highly diluted, will produce marked results—believe, that by trituration, shaking, and diluting an inorganic substance, there is added a *dynanical* or *spiritual* power. So great was Hahnemann's fear lest the *power* or *spirit* developed in an inorganic substance would be too powerful, that he directed that but two shakes be given.

In regard to remedies, little need be said in this connection. An entire change of drugs could not be immediately effected, and to remedy this, those in use (in some cases, at least,) were called by different names. Quinine was called China; Calomel, and the mercurial preparations, Mercurias. Many drugs which had been dropped from the pharmacopœia were taken up and paraded before the public as newly-discovered. Articles of food, which we take daily in variable quantities, and which we were not aware created or cured disease, when properly potentized, and used as directed by Hahnemann, became powerful remedies in this new system. Common salt, used homœopathically, is "a powerful and heroical medicament, which can only be administered to patients with the greatest caution." We inhale the fumes of sulphur to a considerable degree, as when lighting the ordinary match; we are not aware of its creating or curing any disease, yet, carried to a high attenuation, and given by homœopathic hands, it produces, we are told, marked effects. Bryonia is a drug mentioned quite frequently in homœopathic works, and said to be indicated in many diseases. The name is new to many of us who are young in the profession, but, by referring to our old pharmacopœias, we find it, and it is only dropped because its properties are excelled by many of our more modern drugs.

I might multiply these examples, but must pass to another topic.

The question may occur to some, how was Hahnemann able to make such a discovery? How perfect such a system of medicine? How obtain such a profound knowledge of drugs? etc., etc. I answer, mainly by his *provings*. By this, I mean, their experiments with remedies on themselves and their patients. How were these provings made? Mainly by Hahnemann and his disciples; but, in many instances, by honest and conscientious regular physicians. The result of those made by the latter class I shall notice in another part of my paper; what Hahnemann and his followers ascertained should be noticed in this connection.

Hahnemann says, upon the proving of remedies and the classification of the symptoms which arise, "depends the exactitude of the whole medical art, and the weal of all future generations of mankind." At first, the experiments were made with the crude drug, using the ordinary dose, afterwards by observing the effect with infinitesimal globules and dilutions. Hahnemann advised (see *Organon*, p. 218,) the 30″ dilution as the best for proving the power of a drug, and also claims that while under provings, "all symptoms observed are to be attributed to the medicine." What nonsense! Suppose, for instance, ten persons were selected—good, bad, or indifferent; plethoric or anæmic, sick or well; imaginative or of a cooler temperament—or, make the circumstances the most favorable, take ten persons, honest, healthy, and conscientious, not susceptible to nervous tendencies, *scientific*, if you please—give them the crude drug or the infinitesimal dose, and let them understand that every feeling, desire, or bodily inconvenience is to be attributed to the drug; what will you have? Look at this display of symptoms taken from their own works. "After stooping some time, sense of painful weight about the head upon resuming the erect posture." "An itching, tickling sensation at the outer edge of the palm of the left hand which obliges the person to scratch." In the provings of *hydrastis canadensis* we are told (the dose being given at 8.45 a.m.), that at 11 a.m., sneezing in the sun caused flickering in the eyes."

Dr. Hempel calls particular attention to the "provings" made with sulphur. A student having taken for three weeks, three times a-day, five globules moistened with the tincture of sulphur, began to have a very troublesome feeling of fatigue. In the course of a few days the pain was felt deeper, as if in the young man's bones; a short time afterwards the head of the right tibia became very painful, and after the slightest exercise he had to go and lie down." All these terrible pains were caused, it will be remembered, by taking five globules, moistened with tincture of sulphur, three times a-day. The learned Dr. also favors us with some other symptoms caused by taking sulphur: "shooting pains in the eyes," "boring pains in the ear," "raw pain in the mouth," "digging pain in the teeth," "drawing pain in the upper teeth," "sensation as if he were smelling soap suds." He closes his lecture on sulphur with this grandiloquent burst of rhetoric: "If you would conquer the great mind of the profession, then let me urge you to ever think of homœopathy with hearts full of reverence for the consistency and universality of her teachings as a doctrine of life; a heavenly truth, which will not fail, if properly understood and universally applied, to link earth and heaven in one great cycle of sensual refinement, intellectual beauty, and social and religious harmony."

Some of the symptoms produced by common salt are, "rigidity of joints when they are moved," "paralysis," "frightful dreams of quarrels, murders, fires, thieves," etc., "awkwardness," "redness of the great toe"—many others are given. Symptoms produced by chalk: "great tendency to strain the back in lifting," "on walking in the air, sadness with tears," "flaws in the fingers," "disposition to weep, even about trifles," "disgust and aversion to all labour whatever," etc. These are but a few mentioned in Jahr's *Manual of Homœopathic Medicine.*

I have thus, gentlemen, given you an account of some of the leading doctrines of homœopathy as taught by Hahnemann. No attempt has been made to represent them unfairly, and my assertions are mainly from homœopathic authorities.

One doctrine remains to be examined—the law of cure—"*Similia Similibus Curantur.*" When I shall have examined homœopathy, as *now practiced,* you will agree with me in the statement I now make. The law of cure, divested of everything else, either devised by Hahnemann or dragged into the system by some of his followers, is the only disputed point between physicians and homœopaths. I therefore leave this point till near the close of my article.

II. I now come to the second part of my paper—attempts to gain for homœopathy governmental patronage, with experiments by both governments and individuals.

Hahnemann published his first paper in 1796; nine years later his first work; five years after this his *Organon,* and in 1828 his *Treatise on Chronic Diseases.* Hahnemann's ideas were somewhat, yet not entirely, original, and governments and individuals were not slow in testing the merits of his system. Governments are always willing and anxious to improve the hygienic condition within their boundaries, and our leading and best physicians are not biased in their opinions, but willing to avail themselves of everything to combat disease, and alleviate human suffering. The assertion is a false one, that physicians do not investigate new theories of disease. The true physician is investigating constantly, and he incurs the displeasure of such as Hahnemann and his followers because he will not permit himself to be influenced by money, enthusiasts, and pretenders. The Christian clergymen incur the displeasure of such as Joe Smith and Brigham Young for the same reasons. But what Government or individual, it may be asked, ever gave homœopathy a fair trial and found it wanting? Let us look over the list.

"In 1829, by order of the King of Naples,* a commission was appointed to test homœopathic remedies, under the following restrictions:

* See *Chicago Medical Journal* and *American Medical Times.*

"1st. The Commission shall consist of two Professors of the University of the Faculty of Medicine; two members of the Medico-Chirurgical Academy; two members of Public Instruction, and the Heads of the Hospital.

"2d. The Commission, after having proved the attenuations of the homœopathic remedies, shall place the said remedies in a strong box, firmly closed, with two different locks, the keys of which shall be returned, one to the Director of the Clinique, and the other to the Commissioners charged with following the treatment.

"3d. The clinical ward shall have but a single door, guarded by a sentinel; its internal arrangements shall be adapted to health; it shall not contain more than fifteen to twenty beds, and two visiting physicians, one chosen by the attending physician, the other by the Commissioners, who shall keep an exact register of all that happens to the patients, the changes in their disease, their regimen, cures, deaths, if any die.

"4th. The admission of patients affected with acute or chronic diseases, shall be left to the choice of the attending physician and commissioners, with this condition, that the attending physician shall not be obliged to take patients known to be incurable, nor shall diseases equivocal be considered proper for positive experiments.

"5th. The Commissioners having selected the class of diseases, the attending physician shall make known the symptoms, administer the remedies, and prescribe the regimen.

"6th. Each day the condition of each patient shall be determined by the attending physician and commission.

"The result of this trial of forty days of homœopathic treatment, under the observation of the commission named by the King of Naples, was the conclusion, that not only is this treatment of no effect, but that in certain diseases it has the inconvenience of preventing the employment of remedies capable of effecting a cure. The physician in attendance was M. de Horatus, author of a homœopathic work, and who had boasted of the most marvelous cures.

"Clot Bey, Physician-in-chief to the armies of the Viceroy of

Egypt, states (*Annal de la Med.-Physiolog,* Sept. 1834, Ency. Decr. 1834,) that a German homœopathic physician petitioned the Council of Health to try this system in the Hospital of Cairo, alleging its cheapness, etc. He was allowed to select patients suffering from ophthalmia and dysentery. The Council were convinced from this experiment that the homœopathic system was not entitled to their confidence. The following is the conclusion of the report of the Council of Health: "That the cures obtained were due simply to the hygienic and dietetic treatment adopted, and not at all to the infinitesimal doses." So unsuccessful did the trial prove, that the homœopathic practitioner was obliged to abandon the country.

"In April, 1832, a ward of thirty beds, in the Hotel Dieu de Lyon, was placed in charge of M. Guerard, the most distinguished homœopathic physician of that city, with liberty to select his patients. He selected fifteen, suffering from febrile affections—pneumonia, erysipelas, catarrh, etc. He visited them daily, and in presence of sixty students and several physicians, examined, prescribed homœopathic remedies, and directed the regimen. The experiment continued for seventeen days, when the physician voluntarily retired. During this time there was no improvement in the patients, nor any advantage gained which could be ascribed to the homœopathic treatment. The physician attributed his failure to the action of deleterious miasma, always existing in hospitals, and from which he could not protect his patients. He acknowledged that the remedies which produced such powerful effects in private practice, utterly failed in hospitals, owing to the emanations from the bodies of persons collected together, which neutralized the infinitesimal doses.—*Gaz. Med. de Paris, Ency.* Nov. 1833.

"In 1834, M. Andral employed homœopathic remedies in one hundred and forty cases, in the Hopital de la Pitti, of Paris. The arrangements of the ward, the regimen of the patients, and all the details of treatment, were carefully managed according to the direction of Hahnemann. The remedies were all obtained from the most eminent homœopathic apothecary in Paris, and administered with the most religious exactness. The result of this trial proved the entire insufficiency of the remedies employed. It was found necessary, in most of the cases, to resort finally to the regular treatment.—*Bull. Gen. de Therapeut.* 1834.

"In 1835 the Homœopathic Society of Paris petitioned the authorities to establish a Homœopathic Hospital and Dispensary. The Minister referred the matter to the Academy of Medicine, which appointed a commission to draw up a report. This commission reported in substance as follows: That they had submitted the system of homœopathy to the most rigid tests and practice, without obtaining any other than negative results, so far as the action of remedies was concerned; while observation proved that grave dangers were liable to follow its adoption in some diseases, from the neglect of proper and reliable remedies. If the authorities yielded to this request, the advocates of Mesmerism, animal magnetism, etc., were equally entitled to have hospitals opened for the trial of their peculiar systems, and thus every form of quackery would demand attention. They advised that the petition be not granted. The Minister of Public Instruction, acting upon the advice of this report, refused the petition.

"In 1829, the Czar of Russia ordered that the system of homœopathy should be tried in several military hospitals. For several years the practice continued, and reports of marvelous success were annually published, but it has entirely failed of obtaining the confidence of the Government, and by a recent edict it is forbidden to practice homœopathy on the Russian territory."*

* Some months since the *Examiner* published a communication asking information in regard to the practice of Homœopathy in Russia. On inquiry of Col. Croswell, a gentleman of great culture and acute observation, who was appointed United States' Consul to St. Petersburgh by President Buchanan, and resided in Russia some 15 years, I learn, that Physicians from other countries who take up their residence in that empire, and desire to practice Homœopathy or Allopathy, are required to pass an examination before the Imperial (Allopathic) Board of Medicine; that a diploma from a Medical College, well-known and in good repute, has considerable weight as evidence of qualification, and makes the examination less formal; that a Physician is not allowed to practice medicine without obtaining a certificate or license from the Imperial Board. The certificate is issued with the understanding, of course, that the regular sys-

In 1857, a resolution was introduced before the Board of Governors of the Almshouse Depot, New York, providing that one-half of Bellevue Hospital should be set apart for the practice of homœopathy. A committee was appointed to investigate the claims of the system, and in their report many of the facts and experiments brought out in Europe were communicated. I am indebted to the report of that committee, of which Washington Smith was chairman, for much of the preceding. It is not necessary for me to state to you, gentlemen, under whose management Bellevue Hospital has continued to advance and improve. It has been under the charge of gentlemen whose works we delight to peruse, whose names are bright on the page of our medical literature; men of professional standing—men *educated, enlightened, liberal,* but *always* of one school.

In 1862, the United States Senate took up the question, and Senator Grimes, of Iowa, introduced a bill placing some of the hospitals in and around Washington under the charge of homœopathists. For aught I know, the eastern army may have been delayed while this momentous question was being considered. It was found, however, upon investigation, that other governments than ours had been petitioned—those petitions granted—the system tested, and in every instance failed. Our Government thought too much of her citizen soldiery to turn them over to a system so utterly devoid of anything to recommend it. Men, professedly homœopathists, *did* obtain commissions as surgeons in our army, not to practice *homœopathic* surgery, or by passing a homœopathic examination, but by that miserable system of subterfuge and evasion, which, I am forced to say, they practice every day of their lives.

But what have individuals done to test this system? Have honest and competent men not pledged to this particular school ever tested its merits? The remark made in regard to governments always being ready to examine any new theory based on tem of medicine is to be practiced. It is highly probable, however, that after obtaining their license they practice what they please. Perhaps I should say, that Col. Croswell has been treated homœopathically, and is not at all pledged to our system.

science with the desire to improve her hygienic condition, I may now repeat, and apply it to individuals. As a profession, we desire everything that will benefit our patients. We are not given to any one idea, or school, or ism. We are not such consummate fools as to let a patient die, if anything under God's heaven, wherever found, would save that patient. And yet, if one takes homœopathic authority, he would conclude that we plan to let those committed to our care perish.

Some of our most distinguished physicians have tried homœopathy; let me quote from some of them. M. Andral says, "I have submitted this doctrine to experiment; I can reckon at this time from one hundred and thirty to one hundred and forty cases, recorded with perfect fairness, in a great hospital, under the eye of numerous witnesses; to avoid every objection I obtained my remedies of M. Guibourt, who keeps a homœopathic pharmacy, and whose strict exactness is well known; the regimen has been scrupulously observed, and I obtained from the sisters attached to the hospital a special regimen, such as Hahnemann orders. I was told, however, some months since, that I had not been faithful to all the rules of the doctrine. I therefore took the trouble to begin again. I have studied the practice of the Parisian homœopathists, as I have studied their books, and I became convinced that they treated their patients as I had treated mine, and I affirm that I have been as rigorously exact in the treatment as any other person."

This same distinguished physician, assisted by several others in good health, also experimented upon themselves with their most highly extolled remedies. The experiments continued for one year, and were with such drugs as cinchona, aconite, sulphur, and arnica. At the end of the year, he publicly stated to the Academy of Medicine, that not a single symptom attributed to arise from the use of the drug was experienced by either himself or associates.

"During the Peninsular War, M. Bonnet, President of the Royal Society of Medicine, of Bordeaux, used cinchona as a preventive against various diseases, but never noticed the pretended symptoms." It has been claimed that belladonna, given in infinitesimal doses, would prevent scarlatina or cure the disease *instanter*, where the remedy had not been given as a prophylactic.

Many good mothers in our land are undoubtedly waiting, with the omnipotent-globule in hand, the first appearance of this dreaded disease, never doubting its power to cure.

In 1849, two homœopathic physicians in Cincinnati, claimed to have treated 1116 cases of genuine cholera with a surprising small loss; but the superiority of homœopathic treatment of cholera may now be confidently denied. Even the boldest defenders of the system now admit its insufficiency when the disease is severe.

Dr. Russell, one of the editors of the *British Journal of Homœopathy*, says—"We cannot help deprecating the boastful tone we so often hear assumed by homœopathists on this subject—the treatment of cholera." Dr. Fluschman, of Vienna, with a large experience in homœopathic treatment of cholera, says—"Every remedy which has been recommended has been tried and tried again by us, but I have little to say in praise of any of them."

I have now passed in review Parts I and II of my essay. I have examined the leading ideas of homœopathy excepting the law of similia. The trials given homœopathy by different governments have also been given. We have seen that Hahnemann was not a man whom we would naturally expect to be the author of any thing original or remarkable. He conceived a theory and at once declared it—"Infallible." We have spoken of the dose, the manner it is prepared, etc.; we have noticed also the itch doctrine. This system of medicine was given to the world about 75 years ago. How has it stood the test? What does Hahnemannic literature say on the subject?

Examining it fairly, without prejudice or passion, let us see if the practice of the gentlemen of our city, known as homœopathists, agrees with the rules given by Hahnemann. With an earnest desire to speak the truth, let me answer the question. At first I am compelled to say, and our homœopathic brethren confess it, as will be seen by a quotation which I shall read presently, that they have hardly a work written by a homœopathist to which I can refer. There is a perfect dearth of homœopathic authority on every thing except therapeutics.

From the very beginning there has been a gradual letting down from the doctrine of Hahnemann. First they tell us there is nothing in the dose. They use enough medicine to cure the disease, but on the *homœopathic principle.* If they do away with the dose they must of course do away with their fine figured way of preparing it. One by one they throw overboard the pet idea of their patron saint Hahnemann. They say that homœopathy is advancing, Hahnemann's ideas are very old. Dr. Richard Hughes has written a work, purporting to be to a friend (a convert), who is anxious to know something of homœopathy. He devotes 22 pages to mercurius—calls it calomel, lauds it to the skies, and says we give it in certain affections in about the same dose you do, stopping soon enough to avoid salivation. Who don't?

Dr. Holcombe, of cholera fame, has published a little pamphlet, in which he assumes to tell us just what homœopathy is. He advises the use of about everything the regular physician places any confidence in, even the much-abused and neglected lancet, and yet you will all remember that it is on the *homœopathic principle!*

In this city we have about 75 medical men professedly homœopathists. A prominent and influential member of the school informs me that only eight or ten at most of that number are pure undeviating homœopaths. One of our druggists gives it as his opinion that they have only five or six who are true to their profession. These gentlemen, if I understand it correctly, believe every word of Hahnemann's writings, and try to follow his teachings. What shall I say of the rest? Several of them are professors in the Hahnemann school in this city. The word homœopathist is displayed on many of their signs. By every way possible they certainly pretend to be homœopathists. We see them on the street with the customary little pocket cases. Now, how about their practice? It is altogether at variance with what they profess. One of their professors in this city, recently gave for a periodical neuralgia, quinia sulph. grs. xxiv, divided into six powders, one powder every four hours. The patient was cured in 24 hours. This was after about ten days treatment with sugar-coated granules. Another homœopathic gentleman, an author, at least he has written one small work, buys calomel and quinine by the ounce. Some of his prescriptions also are known to some of us; they are simply *immense.* A medical friend of mine vouches for the truth of the statement, that he knows of grs. ij. of quinine being given every two hours by a homœopath to a child ten years old. The same friend was called to visit a child in convulsions, who was under the treatment of a homœopathic gentleman. The family, having always had regular treatment, had only recently employed a homœopathist. They were disappointed in homœopathy and spoke of changing again. The homœopathic physician said to the family—"You need not change physicians; your child is taking allopathic drugs in allopathic doses!" At this very time almost every available place in our city is covered with an advertisement of somebody's "Home Bitters." The dose of the compound, as given on the bottles, contains about 6 grs. of gentian, 3 of wild cherry bark, 9 of orange peel, 3 of camomile, 3 of Peruvian bark, 3 of cardamon seeds, and 6 of columbo. It is endorsed and recommended by five homœopathic professors. The *Investigator* confirms this with lamentations.

Some of the homœopathic physicians in this city, in the treatment of a patient, are accustomed to give large doses, such as 20 grs. bromide potassium, associated with, perhaps, a millionth of a grain of lycopodium. If the patient recovers, it was the lycopodium that influenced the disease. A well known homœopathic physician, with an office in quite a central locality, always appears on the street with two homœopathic medicine cases under his arm. An acquaintance, a clerk at the drug store near the gentleman's office, upon going to a neighboring city to locate, gave me a specimen of homœopathic prescription writing. This physician has his private formulæ for ointments and mixtures, and in addition, gives in the ordinary way, iod. potass., mercury, opium, morphia, quinine, in fact, every remedy, which is considered standard by the so-called allopath, in good round doses. But it is a needless expenditure of time to multiply such testimony, inasmuch as it only establishes what homœopathists themselves are frank to confess.

A few *propositions*, advanced by our homœopathic friends, deserve to be noticed. They tell us that homœopathy is an advance from allopathy. Said a well-known homœopathic physician to me some months ago, speaking of his long continuance in the profession and his advanced age, "I jumped ahead of you fellows 25 years ago; I am a homœopathist." A few words in regard to the claims that homœopathy is any advancement in medicine and I pass to a second assertion. The living body is made up of organs. All are endorsed with certain and appropriate natural powers. These powers are so adjusted that they have a tendency to preserve the whole organism in a perfect state. When, from any cause, internal or external, any irregularity occurs, if slight, this regulating principle is sufficient to control the action of that evil cause, and restore the system to its natural condition. If the cause of the disease is profound, too severe for this regulating principle to regulate, then the physician with his remedies steps in to assist this principle. This I understand is the true system of medicine. The science of medicine has advanced during all past time hand-in-hand with the kindred sciences. The advance has been slow, but the improvement sure. Articles in our materia medica worthless have been thrown out.. There has been a general weeding out. Physicians write less intricate prescriptions; they have learned to trust nature more. And the only principle which I know anything about, homœopathy, eclectics, hydropathy, *et al.*, to the contrary notwithstanding, is that a sick person is to have just what experience has taught will cure him.

Homœopathy came, and with one mighty bound went *back* 500 years. Its advocates invaded the *loathsome* for remedies with which to humbug the people. Take Jahr's Pharmacopœia and look at their remedies. On page 96 you will find an account of the American pole-cat or skunk. The learned author of the advance school says, "near the *anus* there is a pouch where the follicular glands secrete an odorous matter; the animal squirts his liquor, etc. We make the three first attenuations by trituration." The black spider (see page 83) is prepared for homœopathic medication by putting the whole animal in alcohol; "macerating it for weeks, and even months, and then decanting the clear liquor, which is the mother tincture." "The three first attenuations of the common wood-louse" are prepared by trituration; the tincture, by 20 parts of alcohol. "Lachesis (snake poison) is procured from the poison bags which are found in the upper jaw of these reptiles."

Prof. O. W. Holmes writes that, "it is said the *pediculus capitis* (the common head-louse) is actually prescribed in infusion—hunted down in his capillary forest, and transferred to the digestive organs of those he once fed upon."

What may we expect next? The Album Græcum has been omitted. That was formerly used in medicine. Why not prepare the first three attenuations by trituration? And this is advancement!

Again, they say it is *popular*—it is supported by enlightened and fashionable people.

I know at least one homœopath—who, by the way, is quite true to Hahnemann—who has it as a standing argument, that the high-bred, the elegant, the first-class people patronize him. His practice is all good pay. He never has any low Dutch and Irish apply to him for medical advice.

I admit that poets sing its beauties, that dyspeptic clergymen exalt its merits in their papers, and that it is a favorite with babies. There is also a class of delicate, nice, fastidious, squeamish people, who eulogize this system. They panegyrize, cry up, and bless everybody homœopathically inclined. It is so pleasant to have the doctor around twice a day, the medicine is cheap, and then it is *sugar-coated.* And those homœopathic balls—they have had two in New York and one in Chicago—are such high-toned affairs, nothing low or vulgar, none of the poor there, and they do *so much* good. This argument is certainly weak, yet some homœopathic physicians, and many of their patients, use it.

Granting for a moment that it is true—that all the fashionable employ homœopathists. What part of our population live in marble-fronts? How large a part on our avenues? What class of people have acute disease—diseases which need treatment—and what class, aches and pains, which only a homœopathist can enumerate? The poor, the over-worked, those living in unhealthy localities, families not surrounded with those things which make life happy and promote healthfulness in the community; those exposed to every change of weather; the middle-class, constituting the great majority—are the persons who are acutely sick; among these various classes thus enumerated the true physician finds his work.

The pretensions of homœopathy have never succeeded among these people. It sounds badly, however, even if true, for homœopathists to boast and swagger over patients elegant and refined, yet simply dyspeptics and hypochrondriacs.

Our homœopathic friends have a few standard statements, which they are accustomed to throw out to the people at every opportunity. They never let a chance go by without suggesting some of their favorite considerations to those who will listen:—

1st. The distinguished physicians who have embraced homœopathy.

2d. The decreasing death-rate in hospitals as soon as homœopathic treatment is instituted.

3d. Their wonderful cures.

When we come to examine these statements we are surprised at the bare-faced misrepresentations. Hahnemann was accustomed to make a most formidable display of distinguished authorities. Several gentlemen have taken the time, and have tried to look up those authorities—some of his quotations cannot be found—many others, Prof. Jœrg, of Leipsic, has proved to be adulterate and false; and, of the few found by Prof. Oliver W. Holmes, two were wrongly quoted and one a gross misrepresentation.

Among the many names, displayed in an imposing manner, of those who had openly confessed the power of homœopathy, and acknowledged its supremacy over the ordinary method of practice, may be found M. Breschet, of Paris. Prof. O. W. Holmes has a literal translation of a letter from this gentleman, which I will read:—

"*Dear Sir and Respected Professional Brother:*—You have had the kindness to inform me in your letter, that a new American journal, the *New World*, has made use of my name in support of the pretended homœopathic doctrines, and that I am represented as one of the warmest partisans of homœopathy in France. I am vastly surprised at the reputation manufactured for me upon the new continent, but I am obliged, in deference to truth, to reject it with my whole energy. I spurn far from me everything which relates to that charlatanism called homœopathy; for these pretended doctrines cannot endure the scrutiny of wise and enlightened persons, who are guided by honorable sentiments in the practice of the noblest of arts.

I am, etc., etc., G. BRESCHET,

*Prof. in the Faculty of Medicine; Member of the Institute; Surg. of Hotel Dieu., and Consulting Surg. to the King.*

PARIS, *3d November. 1841.*

In regard to hospitals, Hahnemann established one at Leipsic, and yearly issued a report, giving remarkable cures, and proving, by tabulated statements, the superiority of homœopathic treatment. The hospital ceased the year following Hahnemann's death. At least two homœopathic hospitals, one in London and one in Vienna, have been discontinued within the past few years. They complain that the control of hospitals is not given into their hands. When they are established they are not able to sustain them. Tabulated statements, pretensions, and braggadocio will not maintain a hospital. Sooner or later the truth will come to the light.

The ostentatious ways in which they speak of their cures is truly amusing. I can barely notice their fallacies in this respect.

I believe it is universally conceded by all members of the regular profession, that the efforts of nature will, in many instances, sooner or later, cure a great part of the milder diseases we are called to treat. Perhaps in 90 cases out of every 100 in daily practice, our patients would, in time, recover through the restorative efforts of nature. The physician assists these efforts of nature, whose tendency is to cure the patient, and therefore, as a matter of mere economy, he is useful in most of the milder diseases. That man who would be sick ten days if left entirely to himself, might, in many cases, be restored to his business at the end of the second by obtaining the advice of a physician who recognizes the power of nature, and is willing to use a remedy which experience has proven will assist the power already at work.

Not so with the homœopathic physician. He cares nothing for physiology or pathology. He recognizes no power of nature. "*Similia Similibus Curantur.*" A little more of the same poison. And if 90 out of 100 get well, under homœopathic treatment, the little globule *always* does it.

Rummel, a distinguished writer in the homœopathic school, cures a case of jaundice in *thirty-four* days—pulsatilla, aconite, and cinchona are the weapons.

A man in Saxony sprains his ankle, and is so unfortunate as to call an allopathist. He is treated in the common vulgar way for two weeks, and not cured. He calls a homœopathic physician, and, strange as it may appear—*mirabile dictu*—in a little over a month is cured by arnica used homœopathically. This extraordinary case is then published.

Another case is recorded of a patient with a cold in the head. She is taken through that illness, restored to her weeping friends, in six days.

The President of the Society informs me, that a few years ago, the fact was blazoned before the public, that a fractured bone united, actually *healed,* in three days under homœopathic treatment.

It is now going the rounds among our homœopathic brethren in this city, that pulsatilla given in an infinitesimal dose will correct the malpresentation at the superior strait. But reports are nothing, let us proceed on a scientific basis, and take one of their journals for authority. (See *Medical Investigator* for 1869, page 123).

Dr. Beebe, of this city, is speaking in the State Homœopathic Society. "He believed physicians should draw a wide contrast between homœopathic and allopathic surgery. There is a vast difference in the result. The mortality is very much less, and we can undertake operations with hopes of success that they dare not undertake. So sure is he of success, with his remedies to aid him, that he feels able to undertake almost any operation. Believed that before another twelvemonth he would extirpate a lung. Has a case now, were it the right lung that was diseased, instead of the left, he would excise it."

The axiom, *Similia Similibus Curantur*, I shall notice very briefly. This, it appears to me, after a very patient review of the way homœopathy is actually practised in Chicago, is the only disputed question. If there is to be any discussion between the two schools, it can be on this question, and this only. The infinitesimal dose has long since been given up. The high attenuationists sometimes attempt to argue, from a Hahnemannic standpoint, or perhaps, at this time, they would quote Grauvogl, who clearly is now their champion. But the high potency men are so few and far between (about two per cent. of practicing homœopathists) that the mass of homœopathists controvert their argument at once. It is difficult to argue a question which they openly deny in practice. The mass of practicing homœopathists give as large doses and the same remedies as the regular school, and they dare not deny the fact. The assertion that it is all done on the homœopathic principle is too wide a departure from the truth for any body to believe You must then count out 98 per cent. of practicing homœopathists—set them apart, and label them *mongrels*, The remaining two per cent. must prove—

1st. That small doses of a drug will ALWAYS cure a disease, which, when given in larger doses, will produce the symptoms of that disease.

2d. That a drug which will not produce similar symptoms *will* NEVER cure a disease.

3d. That a molecule of an organic or inorganic substance, by trituration and shaking, becomes equal to a molecule of *living germinal* matter.

4th. That a molecule of this living germinal matter, homœopathically prepared, and enveloped in a covering of sugar, introduced into the mouth and down the œsophagus, will go directly to the diseased germinal point, and by *dynamics*, or *spirit power*, heal it.

5th. It will be incumbent on them to explain why so many who profess homœopathy get so far away from what they believe.

If they have such powerful remedies which will produce the desired result on the principle of *similia*, why in the world don't they use them? Why use our *crude poisonous* drugs when they have such elegant sugar-coated preparations, at once *so powerful*, and yet never known to injure any body? Let us hear from some of them on these points.

I come now to the last division of my subject. I must not omit to speak to you of a new text-book on homœopathy—Grauvogl's new book.

In the *Homœopathic U. S. Medical and Surgical Journal*, for January, 1867, will be found a most urgent and no less humiliating demand for the production by homœopathists, of standard medical works. It shows as well as anything I could write the kind of men who have embraced homœopathy. It is the confession of homœopathists themselves, contained in their own language, as follows:

"A leaden apathy has for a long time past been upon our homœopathic physicians East. We want solid acquirements everywhere; we want in our schools more pathologists and learned physicians—as Bennett, Watson, and a score of others. Writers, for instance, upon female diseases, and their surgical and mechanical treatment; and writers on obstetrics, such as Bennett, of London, Sims, Simpson, and Barnes. When will issue from our ranks, writers of such worth as Rayer, Casenave, or Wilson on diseases of the skin? Louis Andral, and Skoda, on diseases of the chest—West on diseases of children—Ricord on syphilis; or such pathologists as Rokitansky, Virchow, or Rock? Homœopathy is here a *humiliated beggar* to allopathy. Produce—produce! Were it but the pitifulest infinitesimal fraction of a product, produce it, in God's name!"

This is truly a homœopathic wail—a modern and infinitesimal cry for learned and educated men to write homœopathic books. "When will issue from our ranks," he cries in perfect despair. While the above pathetic appeal was being written, a gentleman in this city, noted for his charities, and, from all I can learn, a conscientious truthful homœopathist of the pure unadulterated Hahnemannic school, was translating a work from the German. The book was issued from the publishing house of the Western News Co., in the fall of 1870. Judging from what the homœopathists say of it, one must conclude at once that Grauvogl is the most candid, the most thoughtful, the boldest, the most enthusiastic, and the *only* writer of the age, and his book destined to supply at once the great want of standard works which has heretofore existed in every department of their entire science. The book is before us, containing nearly 800 pages of reading matter, well bound, with a picture of the author. The *Chicago Times* speaks of it carefully—praises its typographical excellence, and says the homœopathists are an indomitable lot of fellows. The *Tribune* praises its typography, and predicts that it will soon find a place in every homœopathist's library. The *Brooklyn Daily Union* says, "The work of Dr. Grauvogl has a double significance, as a new contribution to medical science as a whole, and as an exposition of a system which it develops and illustrates with fresh facts and carefully attested analogies." *The Medical Investigator*, Chicago, says, "Grauvogl has created a sensation." Dr. Eggert, of Indianapolis, says, "The work will—nay it must—become the bulwark of our school in the face of our enemies." Dr. Pearson, Mt. Pleasant, Iowa, says, "Grauvogl has opened a masked battery, and it will be difficult to come within range of his guns without being forced to surrender." Dr. Gatchell, speaking of the old school, says, "They will be impressed with the singular ability with which Grauvogl handles their leaders and smashes their idols." Dr. Samuel A. Jones, of New Jersey, speaking of the book, likens it to a sword—"A scimatir, *cleaving* its way, etc." When Dr. Hering read the book, he exclaimed with delight, "At last we have a thinker!"

. Now, gentlemen, you are expecting something formidable. Evidently Grauvogl thinks he has sounded the death-knell of physiological medicine. It is not my province to review this work, but, in order to bring down the history of homœopathy to the present time, I must notice its prominent features.

Text-book of Homœopathy, by Dr. v. Grauvogl, of Bavaria. Translated from the German, by Dr. Shipman, of this City Published by Halsey and the Great Western News Co.; Contains about 800 pages, printed and bound in an elegant manner; the Table of Contents is very copious, and the work is divided into parts I and II. Part I discusses Theories and Principles, Part II the Practice of Homœopathy.

In his introduction, Grauvogl attempts to account for what he terms "the lamentable misconception and persecution of homœopathy." The physiological school, which he very kindly calls us, believe everything *impossible*, which to them is *incomprehensible.* A furious war was waged against the theory of gravitation on account of its incomprehensibility. It has been so at the commencement of all the sciences—homœopathy is not an exception; in its commencement it must expect to be persecuted; and people are acting in the same manner towards homœopathists as they did towards Newton, Galileo, Copernicus. None of the unlearned, and but few of the learned, can comprehend homœopathy. And our homœopathic friends lay themselves down in their little beds and comfort themselves with the idea that they are martyrs to science.

Our author gives his definition of life, health, and disease. It is nothing new for him to advance a definition of life; several have done it before him, and really he does not seem to have given a more correct definition than others before him.

Two men have been selected by Grauvogl as special objects of attack, Virchow and Leibig. With regard to Virchow, he says, page 38, "What he (Virchow) teaches about the cell, as the elementary part of life, are facts of direct perception which constitute the *extreme limit* of that kind of knowledge of the physiological school. But this does not suffice for therapeutics at all, however it may satisfy physiology. He nowhere gives us any intimation touching the chemical composition of the cell." Dr. Grauvogl certainly shows great ignorance when he quotes Virchow as the only authority on elementary structure. He would have us believe that none but Virchow in our school had ever searched for the ultimate atom, and that it was left for homœopaths (especially and mainly to Grauvogl) to discover the only true anatomical unit. He seems to ignore totally Fallopius, Haller, Schwann, Henle, Huxley, and Beale. He would have one believe, and it is so represented in some of the non-scientific reviews, that all the physiological school know about microscopic anatomy is taught by Virchow, and that we accept his teachings *in toto.* We have had fifty men investigating this subject where our homœopathic friends have had one—in this branch of the science, as well as all others, homœopathy has been a "humiliated beggar" to the physiological school.

It is *extremely difficult* for Dr. Grauvogl to understand anything which Grauvogl did not devise. It delights him to speak of "new and far-reaching discoveries," "of gross errors," of such-and-such a thing which "*I* (Grauvogl) was the first to point out." His egotism is beyond all comparison—it must disgust his most ardent admirers. At page 398 we find the following modest sentence: "The hollowness of the physiological school manifests itself as soon as its doctrines are analyzed as has been done in *this work.*"

From page 318 to 381 he gives clinical cases. Grauvogl's patients always come to him after being treated by some one of the physiological school—as a general thing they have been given up to die. Grauvogl *always* cures them—not a single fatal case can I find recorded in his work. At page 158 commences a report of a case. I hope, gentlemen, that I shall not be deemed prolix if I transcribe a considerable part of it.

The patient was 40 years old, and had taken a cold; was weak in his limbs, and was incapacitated for work. On the 7th day Grauvogl took charge of him. Whatever might have been indicated, homœopathically, could not be given, for Grauvogl had no homœopathic remedies at his disposal. He had to take crude allopathic drugs and dilute them in common vulgar water, in order to give any medicine at all. On the 19th day we find the patient in this most remarkable and critical condition. The temperature began to sink—the pulse to increase in frequency till it reached 130; on the morning of the 23d day the temperature was 35.6 C.; the pulse became small, until finally it could not be counted; an erysipelatous bed-sore had formed on the left shoulder, the sacrum, and the two hip-joints. Now I quote *verbatim* from page 161: "The patient, fatally ill, had the following symptoms which the adherents of the physiological school cannot explain—constant trembling of the hands; utter insensibility of the body, except a sensitiveness to a lowered temperature, even from removing the clothes when dressing the bed-sore; the bed-sore on the sacrum became black and hard; the countenance of the patient was rather yellow; his cheeks less red; he lay in a profuse sweat, which stood on his face in drops; at night he was very much excited, and murmured constantly. * * * His deafness was complete; the sense of smell was blunted; the lips and nails were blue; the tongue was dry and hard as a chip, as were his teeth; speech was impossible; although he took the drink offered him as if involuntarily, though greedily, yet he clearly refused it when it was not sweetened with sugar; he was most fond of plum sauce, though swallowing with difficulty." etc., etc.

The symptoms enumerated are all italicized in the translation, and, I suppose, important. Concerning them Grauvogl further says: "What now? What allopath would not be utterly helpless in such a case, and declare the patient lost beyond all doubt? The homœopathist does not give up any patient as long as he still breathes, for the higher the difficulties rear their heads the more earnest is his zeal for the delivery of the patient. The consciousness of his superior power holds him up; a power which rests upon the law of similarity; upon a law of nature; upon a rock, whence flow thousands of healing springs."

Who shall speak after such a gushing quotation? But the man lived. Grauvogl, unfortunate man, could not get to a homœopathic pharmacy, and he therefore mixed ½ gr. argent nitras in 4 ounces of water, and 5 grs. of quinine, with 5 drops of acid, sulph. dil. in 5 ounces of water—a spoonful of each was given every four hours. Of course, the medicine cured him. Great is v. Grauvogl!

In regard to the cause of tuberculosis, our author speaks with great certainty. On page 275 he says: "With the rarest exceptions, tuberculosis is produced by the ossification of the cartilage of the first rib." To some of our older men, who have made the subject of lung difficulties a life work, it will be news that a congenital and hereditary influence, climate, and various diseases have nothing to do in causing tuberculous deposit, but that, with the rarest exceptions, the disease is caused by the ossification of the first cartilage. Dr. Grauvogl, it will be noticed, speaks very *decidedly*. Grauvogl himself makes the statement.

It would have been well for the foremost of writers on homœopathy, since the days of Hahnemann, to have been a little less positive in some of his assertions. He desires to make the distinction between the two schools very marked, and in his ardor certainly makes many statements inexcusable, unwarrantable, and *false*.

Speaking of sun-stroke, on page 270, he says: "That physicians of the physiological school persistently, even to this day, prescribe general bloodletting." *This is not true*, and Grauvogl knows it, or ought to have known it before he wrote this book. Our authors and teachers are explicit on this point. General bloodletting is absolutely contraindicated, except in the apoplectic form of sun-stroke.

The work is filled with misrepresentations, and any one reading the book understandingly and impartially cannot fail to see them.

The author is a very unscientific writer. He makes mooted points indubitable facts, and contradicts himself and the school he represents again and again. On page 100, Part II, he declares, "that nothing which looks like a drug is allowed to be used," while on the following page he gives five rules where "*palliatives*" are permitted if homœopathy fails:

1st. He recommends tinct. opium to relieve pain, in from 3 to 40 drop doses.

2d. Emetics and castor oil sufficient to clear the intestinal canal.

3d. Iodide of potassium.

4th. The so-called diuretics in dropsy.

5th. Alkalies for neutralizing acids.

With one more quotation I am done with Grauvogl. On page 214, in speaking of the dose and remedy, I find the following extraordinary sentence: "The sole and simple question can only be what quantity of a substance, regardless of all subjective, convictions, and incomprehensibilities, is necessary in order to induce that chemical or physical counter-motion in any desired part of the organism, which is equal in intensity, and opposite in direction, to that which is induced by the morbific cause, in order to check this latter forthwith, or, at least, delay it, and then, by repetition, to remove it."

Now, here is a sentence taken *verbatim* from Grauvogl's book. What does it mean? Whatever its import, we must consider it as authority, for it is the utterance of a commander who "handles our leaders and smashes our idols." To me, *simply* and *only* this is meant. A patient has erysipelas, small-pox, or pyæmic fever. Such a dose of a certain remedy should be given (say nothing of the quantity) as will induce a chemical change in the specific poison of those diseases. In other words, neutralize the poison. Why was it necessary for Grauvogl to write 800 pages to tell his homœopathic friends this? It is taught in every college of the physiological school. We practice it every day of our lives. He says it must be "*equal in intensity.*" Certainly it must, or it would not neutralize all the poison. He says, also, "*opposite in direction.*"

How *opposite*, if homœopathic? If opposite, where comes in *similia*? Yet Grauvogl assures us that this *is* homœopathy.

In the first part of my essay, it was shown that with a great majority, at least, of homœopathists, their practice does not differ from ours. Examining the quotations which I have made from Grauvogl carefully—divesting them of much which is superfluous, it is very difficult to find that he has discovered any new fundamental principles of medical science upon which the practitioner can rely in his daily practice, or upon which he can ever hope to establish a practice essentially different from that of the physiological school. He advances the same specious theories—makes the same unwarrantable pretensions, and surrounds all with the same halo of reflected light, as all of his predecessors, but he has not searched out any safe ways in which to walk, or new and firm foundation upon which to stand. His theories, with all of his class, are surrounded with much which appears new. They have made, and are now making, great efforts to harmonize their pet theories with the general laws of our being, respecting which there can be no difference. It has been utterly impossible for them to dose, and slowly but surely they have come back to nearly the point of their departure. After reading Grauvogl it is impossible for us to come to any other conclusion.

This then is HOMŒOPATHY.

